# A General Time-Periodic Driving Approach to Realize Topological Phases in Cold Atomic Systems

**DOI:** 10.1038/srep16197

**Published:** 2015-11-06

**Authors:** Zhongbo Yan, Bo Li, Xiaosen Yang, Shaolong Wan

**Affiliations:** 1 Institute for Theoretical Physics and Department of Modern Physics University of Science and Technology of China, Hefei, 230026, China; 2 Beijing Computational Science Research Center, Beijing, 100084, China; 3 Department of physics, Jiangsu University, Zhenjiang, 212013, China

## Abstract

For time-reversal symmetric cold atomic insulating systems, it is found that the usual driving approach based on electromagnetic field used in solid state systems loses its power to drive them from trivial regimes to topological regimes if the driven systems still hold time-reversal symmetry (TRS). For such systems, we point out that simply varying the optical lattice potential periodically provides a general and effective way to drive them into topological regimes without breaking their symmetries. Based on this approach, we find that the time-reversal symmetric Kane-Mele model can be effectively driven from the trivial phase to topological phases named as Floquet Quantum Spin Hall insulator. Due to the existence of two gaps in the Floquet system, this novel state of matter can stably host one or two pair of gapless helical states on the same boundary, which suggests this state is not a simple analog of the Quantum Spin Hall insulator. This new driving approach to a system without TRS is also investigated.

In the past few years, the theoretical predictions^1^,^2^,^3^ and the experimental observations^4^,^5^ of topological insulator have stimulated strong and continuous interest in predicting new materials and systems with topological phases due to their potential application in spintronics and topological quantum computation^6^.

Topological phases exist in every dimension^7^,^8^, however, it is found that the number of real static systems with topological properties is quite limited. This limitation triggers new proposals to engineer systems with topological properties. One such proposal that time-periodically driven systems can host topological characteristics, the so-called Floquet approach^9^,^10^, recently has attracted great attention^11^,^12^,^13^,^14^,^15^,^16^,^17^,^18^,^19^,^20^,^21^,^22^,^23^,^24^,^25^,^26^,^27^,^28^,^29^,^30^,^31^,^32^,^33^,^34^,^35^,^36^, and has been demonstrated by the direct observation of protected edge modes in photonic crystals^37^,^38^. The great interest arisen are not just because this approach can drive a topologically trivial system to be topologically nontrivial, but also because the driven systems can exhibit unique topological properties without an analog in static systems^39^,^40^, such as the Floquet Majorana fermions with quasienergy *ε* = *π*/*T*^41^.

For solid state systems, currently the general way to drive the system from a trivial phase to a topological phase is by introducing a time-periodic external electromagnetic field to the original static system, like shining light on a conventional insulator. For cold atomic insulating systems, as atoms are neutral, an electric field has no effects on the systems, only a magnetic field can effectively couple with the systems through the spin degrees. However, for an insulator with time-reversal symmetry (TRS), we find that a time-periodic uniform magnetic field can not drive the system from trivial phases to topological phases if the driven system still holds TRS, in other words, no topological phases with TRS emerge through this approach. Fortunately, cold atomic systems are flexible to tune. The most flexible parameter of cold atomic systems to tune is the strength of lattice potential. We find that just simply varying this parameter periodically provides a general and effective way to drive systems into topological regimes without breaking symmetries of the original static systems. As a result, for an original static insulator with TRS, the edge states driven up through this approach are always helical, the same as the Quantum Spin Hall (QSH) insulator^1^,^2^,^3^ (for a TRI superconductor or superfluid, the picture is similar and therefore we restrict ourself to insulator in this work). For the sake of accuracy and discussion, here we name systems with spin conservation and such driven-up helical edge states as Floquet Quantum Spin Hall (FQSH) insulator.

## Results

### Time-reversal symmetric Model driven by varying the lattice potential periodically

We consider a cold atomic realization of the time-reversal symmetric Kane-Mele model in a hexagonal optical lattice. The Hamiltonian is given by^1^


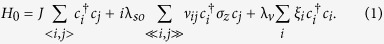


The first term denotes the nearest-neighbor hopping process. The second term is the mirror symmetric spin-orbit interaction which involves spin-dependent next-nearest-neighbor hopping. *v*_*ij*_ takes value 1 (or −1) when the path *i* → *j* is contourclockwise (or clockwise). *σ*_*x*,*y*,*z*_ are Pauli matrices acting on spin space. Pauli matrices acting on sublattice space are denoted by *τ*_*x*,*y*,*z*_. The third term is a staggered sublattice potential (*ξ*_i_ = ±), which are included to control the phase. As [*σ*_*z*_, *H*_0_] = 0, *σ*_*z*_ is conserved.

For a hexagonal lattice, the optical lattice potential takes the form^42^,^43^


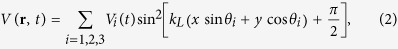


where *θ*_1_ = *π*/3, *θ*_2_ = 2*π*/3 and *θ*_3_ = 0. *k*_*L*_ is the optical wave vector. Here we consider a hexagonal optical lattice with isotropic driving, *i.e*., 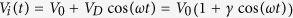, where *γ* = *V*_*D*_/*V*_0_ is a dimensionless quantity. This is like stretching and compressing the lattice along the perpendicular direction periodically, which is different from shaking the lattice along the horizontal direction^29^,^30^ (the mechanism of shaking the lattice along the horizontal direction is to hybrid two close spin-independent bands of certain lattice structure to induce a topological phase transition and consequently the resultant topological phase is of quantum Hall nature which means that the time-reversal symmetry is broken). With such a driving, the hopping amplitudes correspondingly vary with time periodically. When *γ* is a small quantity, we can make an expansion of the hopping amplitudes on 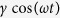 and make the *first order approximation*, then the hopping amplitudes are given by the form: 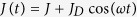, 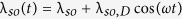, where 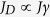 and 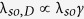 (more details about the expanding parameters and the validity of the first order approximation are given in the Methods section). Then the time-dependent Hamiltonian can be decomposed as 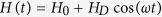. *H*(*t*) is time-periodic and *H*_*D*_ is given by


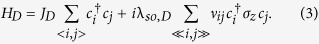


*H*_*D*_ has the same form as *H*_0_ except the absence of a corresponding term to 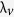 (the inclusion of such a corresponding term does not affect the following conclusion). As 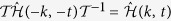, with 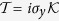, the total Hamiltonian still holds the TRS (see the Methods section).

The single-particle Schrödinger equation associated with this time-dependent Hamiltonian is:


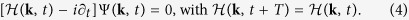


where 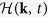 is the form of *H*(*t*) in momentum space. According to the Bloch-Floquet theory, the wave function satisfying Eq.(4) can be expressed as 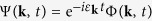 with the Floquet states 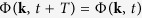 and the Floquet equation 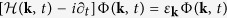. The parameter *ε*, called the quasienergy, is uniquely defined up to integer multiples of 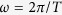. Similar to the crystal momentum of a system with discrete translation symmetry, the quasienergy can be thought of as a periodic variable defined on a quasienergy Brillouin zone 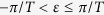.

Although there are many different (but equivalent) ways to compute the topological invariant for a time-reversal symmetric static insulator^44^, to the best of our knowledge, a direct way to calculate the topological invariant for a time-reversal symmetric driven model is lacked until recently^45^. To determine the topological property of the time-dependent Hamiltonian, here we use the ‘repeated zone analysis’^40^. The first step is to expand the Floquet states, 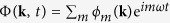. The coeffcients 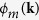 satisfy the time-independent eigenvalue equation


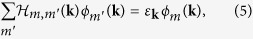


where the matrix form Floquet Hamiltonian 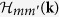 is given by


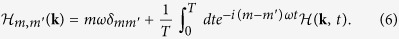


Write more explicitly,


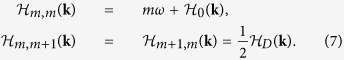


The matrix 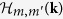 has the block tridiagonal form, where each block is a 2 × 2 matrix.

According to the bulk-edge correspondence, the absence or appearance of edge states traversing the gaps reflects that the system is topologically trivial or topologically nontrivial, respectively. To see whether the driven system hosts edge states, we consider the system with periodical boundary condition in *x* direction and open boundary condition in *y* direction (both zigzag geometry and armchair geometry are considered).

**Figure 1:**
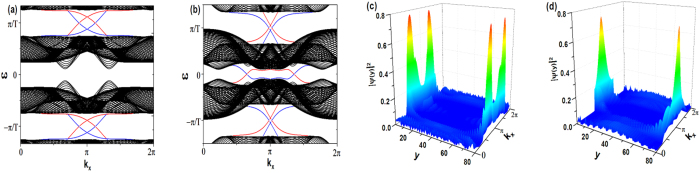
(**a**,**b**) Spectrum of the truncated Floquet Hamiltonian (Eq.(6)) in the zigzag geometry. The parameters used in (**a**) are *J* = 1 (energy unit), 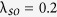, 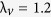, *J*_*D*_ = 1, 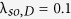, *ω* = 3. The parameters in (**b**) are the same as (**a**) except now *ω* = 2. (**c**,**d**) show the density of the edge states traversing the two gaps of (**b**). (**c**) The density of the edge states traversing the gap at *ε* = 0. (**d**) The density of the edge states traversing the gap at 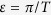.

First, we consider the zigzag geometry. In Fig. 1(a), the static parameters are chosen as *J* = 1, 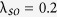, 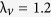 and the next-nearest neighbor distance *a* is set to 1. As 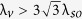, the static model describes a trivial insulator^1^, then according to the bulk-edge correspondence, there is no edge state localized at the open boundary. With the introduction of periodically driving, we find when the driving frequency *ω* is much larger than other energy scales, there is a large energy gap between Floquet bands and no edge states traversing the gap. By decreasing the driving frequency, we find the gap at 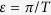 will close and then reopen, with edge states emerging and traversing the reopened gap as shown in Fig. 1(a), which suggests the Floquet band now is topologically nontrivial. The edge states are not chiral since each edge has states which propagate in both directions. As the Hamiltonian holds TRS, the edge states are helical in the sense that fermionic atoms with opposite spin propagate in opposite direction, therefore, the system now is a driven QSH insulator. We name such driven QSH insulators as FQSH insulators. The helical edge states traversing the gap at 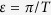 is a unique property of driven systems. For a static system, the helical edge states always appear in the gap at *ε* = 0 because the spectrum of a static system is bounded^40^.

For the sake of discussion, we introduce two *Z*_2_ topological indices *v*_0_ and *v*_*π*_ which both are protected by the TRS to characterize the topological properties corresponding to the gap at *ε* = 0 and *ε* = *π*/*T*, respectively. The topological index taking value 0 or 1 corresponds to the absence or appearance of the helical edge states, respectively. With this introduction, the trivial phase in the large driving frequency region is characterized by 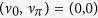 and the FQSH insulator exhibited in Fig. 1(a) is characterized by 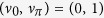.

With a further decrease of the driving frequency, the gap at *ε* = 0 will also close and then reopen. As a consequence, helical edge states traversing both gaps at *ε* = *π*/*T* and *ε* = 0, and therefore, there are two pairs of helical states propagating on the same boundary. Such “anomalous” edge states are without an analog in static system. In static QSH insulator, when there are even pairs of helical edge states, the edge states are no longer stable against disorder and one can always add some extra terms to gap all of them, as a result, the system is topologically equivalent to a trivial insulator. However, for the FQSH insulator characterized by 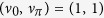, the helical edge states traversing the gaps at *ε* = *π*/*T* and *ε* = 0 are separated by a big energy difference, as a result, their coupling effects can be neglected, and the two pairs of helical edge states are still stable against disorder. Figure 1(c,d) show that these helical states are well localized at the two open boundaries of the system.

**Figure 2:**
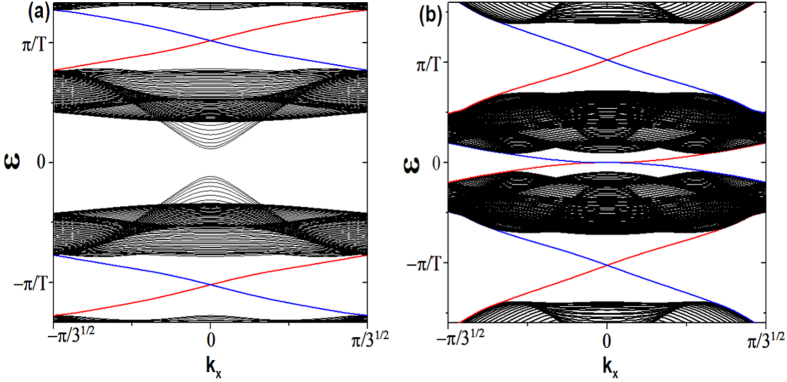
(**a**,**b**) Spectrum of the truncated Floquet Hamiltonian (Eq.(6)) in the armchair geometry. The parameters used in (**a**,**b**) are the same as the ones in Fig. 1(a,b), respectively.

When the zigzag geometry is changed into the armchair geometry but with all parameters fixed, it is found that the edge states appearing in Fig. 1(a,b) also show up, as shown in Fig. 2(a,b). The geometry-independence of the emergence of the edge states suggests that the system indeed undergoes topological phase transitions.

### The effects of a harmonic trap

**Figure 3:**
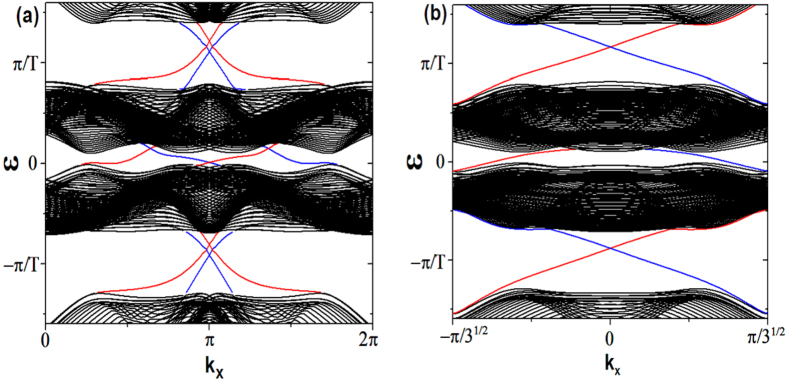
(**a**,**b**) correspond to the spectra of the truncated Floquet Hamiltonian (Eq. (6)) in the zigzag geometry and armchair geometry, respectively. The parameters used in (**a**,**b**) are the same as the ones in Figs 1(b) and 2(b), respectively. The potential of the harmonic trap: 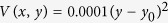, where *y*_0_ is the center of the lattice in the *y* direction. *L*_*y*_ = 80 for both (**a**,**b**).

As real cold atomic systems are always accompanied with a harmonic trap, it is worth investigating its effects. When a harmonic trap is added in the *y* direction (here we only consider a *y*-direction harmonic trap to guarantee the existence of translation symmetry in the *x* direction for the purpose of reducing the difficulty in calculation, however, the following conclusions are also satisfied by the general case), a direct consequence is that the accidental particle-hole symmetry (due to half-filling, *i.e.*, *μ* = 0) of the Kane-Mele model is broken, which is reflected by the fact that the energy spectra are no longer mirror-symmetric along the *ε* = 0 line. However, if the harmonic trap is sufficiently weak (the potential of the harmonic trap at the edge of the lattice system is not larger than the gap), it is found that the in-gap edge states still robustly exist, as shown in Fig. 3(a,b). As here the gap at *ε* = *π*/T is larger than the one at *ε* = 0, it is found that the edge states traversing the gap at *ε* = *π*/T is more robust against the harmonic trap than the edge states traversing the gap at *ε* = 0. When the harmonic trap is strong, it is found that the bands will greatly mix with each other and consequently the edge states will be destroyed by other lower-energy excitations. This can be easy to understand from the local density approximation: the chemical potential 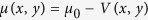 (*μ*_0_ is the chemical potential at the center of the trap, V(x, y) is the potential of the trap). Under this approximation, the energy bands can be approximately denoted as 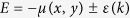. For a strong harmonic trap, the variation of 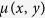 will be larger than the energy gap between the 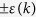 bands. As a result, no matter what in-gap reference energy we take, there will exist low-energy bulk excitations, and consequently the edge states will be destroyed.

### Time-reversal symmetric Model driven by a time-periodic uniform magnetic field

Without loss of generality, we consider a time-periodic uniform magnetic field perpendicular to the plane, whose form is given as


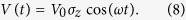


Although the form of this driving term is the same as the one in ref.^10^, the spaces on which the Pauli matrix 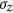 acts are different. In ref.^10^, 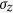 acts on band space, which is equivalent to 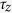 acting on sublattice space here, in other words, the driving term given in ref.^ 10^ is equivalent to a driving term of the form 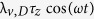 which we have checked can effectively drive the system from the trivial regime to the two topological regimes shown in the previous section. But here 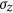 acts on spin space, as we will show, this difference will have important consequence.

**Figure 4:**
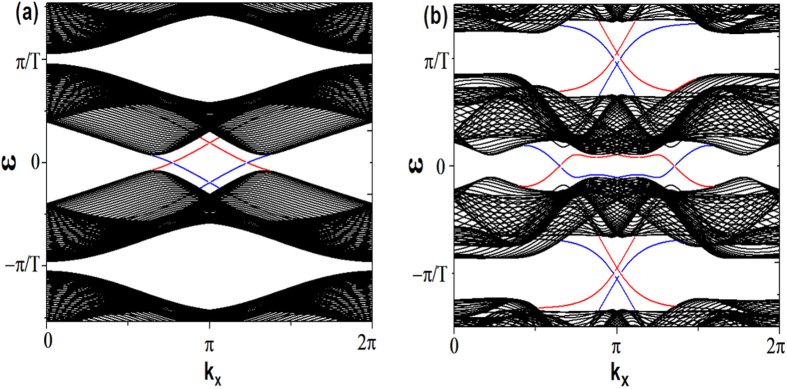
(**a**,**b**) Spectrum of the truncated Floquet Hamiltonian (Eq. (6)) in the zigzag geometry. The parameters used in (**a**) are: *J* = 1, 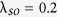, 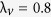, 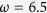, 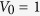. As 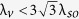, the static system is a QSH insulator, with helical edge states traversing the gap. The parameters in (**b**) are: *J* = 1, 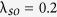, 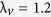, 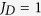, 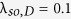, 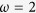, 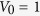.

If the total Hamiltonian is given as 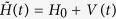, in other words, the system is purely driven by the time-periodic uniform magnetic field, it is found that no matter how strong the magnetic field is, the original time-reversal symmetric static system can not be driven into a FQSH insulator, but it also does not affect the original edge states of a static QSH insulator even it appears as a magnetic field, as shown in Fig. 4(a). The robustness is due to the fact that even the Hamiltonian at any given time may not possess TRS, the Floquet Hamiltonian possess the TRS as long as the condition 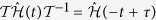 holds for some fixed *τ*^10^ (here 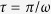. A direct proof is given explicitly in the Methods section). The persistence of the TRS is the reason why the helical edge states are not gapped out.

If 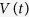 is considered as a perturbation to the Hamiltonian 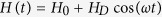, it is found that with the constraint that *ω* is the largest energy scale to guarantee the validity of the Bloch-Floquet theory, no matter how strong the periodic magnetic field is, it also does not affect the edge states of a FQSH insulator, as shown in Fig. 4(b). Note that due to the simultaneous existence of three driving terms in this case, the aforementioned criterion for TRS: 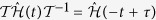 for some fixed *τ*, can not be satisfied. As the system obviously holds TRS, this may suggest that when there exist several driving terms, the criterion for the Floquet system to hold TRS needs to be more relaxed.

When the time-periodic uniform magnetic field is not perpendicular to the plane of the optical lattice, but along some other directions, it is found that the same conclusions are obtained: no matter how strong the periodic uniform magnetic field is, the original time-reversal symmetric static system can not be driven into a FQSH insulator, and all edge states are robust against it.

As general topological insulators with TRS do not need the conservation of spin, to make the study complete, it is necessary to address the question whether the time periodic uniform magnetic field can drive systems without spin conservation into topological regimes or not.To reach the goal, we introduce a Rashba spin-orbit coupling of the form 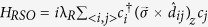 into the Hamiltonian (1)^2^. Then the spin is no longer conserved, but the TRS is still preserved. It is found that by varying the value of lattice potential can also effectively drive the new model into topological regimes where one pair or two pairs of helical edge states stably exists at the boundaries, but the time periodic magnetic field is still incompetent to change the topology of the system.

The incompetence of the periodic uniform magnetic field can be explained by the fact that the topology of the system is in fact only related to the two degrees of sublattice (the energy gap is opened due to the simultaneous appearance of terms with 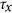 and *τ*_*z*_), it is irrelevant to the two degrees of spin. Therefore, as the uniform magnetic field only couples with the spin degree, its incompetence in changing the topology of the system is natural. In fact, as the irrelevance of the spin degree to the close and open of the energy gap is a fact for all topological insulators with TRS, we can expect that the time-periodic uniform magnetic field is incompetent to drive all trivial insulators with TRS to be topological.

### Time-reversal symmetry breaking Model driven by varying the lattice potential periodically

To see whether periodically varying the strength of lattice potential can also drive a trivial system without TRS to be topological, we consider the familiar two-band tight-banding model realized on a square lattice^46^,


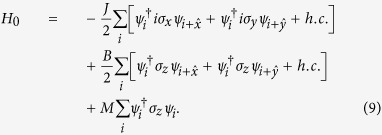


Here 
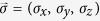
 are Pauli matrices operating on spin, *J* denotes the strength of spin-orbit coupling, *B* and *M* denote the difference between the two spin degrees’ hopping amplitude and on-site energy. Without loss of generality, we assume *B* > 0 in this work.

Re-expressing the Hamiltonian under the representation 
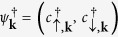
 in momentum space, we obtain


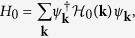


with


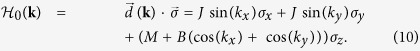


This Hamiltonian is obviously without TRS, consequently, when 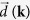 does not vanish in the Brillouin zone, the topology of this static Hamiltonian is determined by the first Chern number^47^,


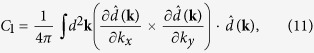


where 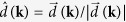, and the Chern number of this system is


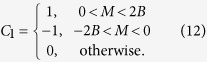


By varying the optical lattice periodically, the parameters appearing in Eq. (9) will also vary with time periodically. Similar to the former TRI case, in the first order approximation, 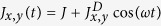, 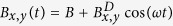 and 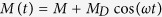. If we assume the two bands are close, 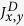 can be much larger than 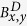, 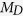. Therefore, without loss of generality, we neglect 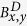, 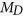 for simplicity, then the time-dependent Hamiltonian is given as


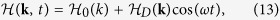


where 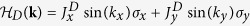. The effect of varying the optical lattice potential is equivalent to varying the spin-orbit coupling periodically.

For the isotropic driving case, 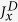 is equal to 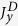. To see how the driving affects the topology of the system, we also consider the system with periodical boundary condition in *x* direction and open boundary condition in *y* direction.

**Figure 5:**
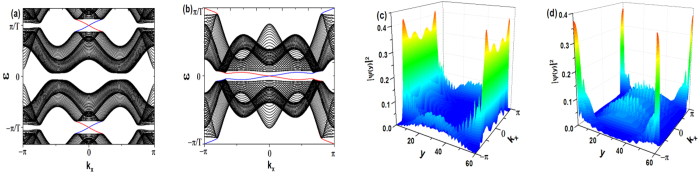
(**a**) Floquet spectrum with parameters: *J* = 1, *B* = 0.2, *M* = 0.5, 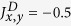, and *ω* = 1.5. (**b**) Floquet spectrum with parameters: *J* = 1, *B* = 0.3, *M* = 0.8, 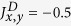, *ω* = 1.3. (**c**,**d**) show the density of the edge states traversing the two gaps of (**b**). (**c**) The density of edge states corresponding to the gap at the quasienergy *ε* = 0. (**d**) The density of edge states corresponding to the gap at the quasienergy *ε* = *π*/*T*

Based on Eq.(12), the parameters in Fig. 5(a) suggest the system in static is a trivial insulator without edge states. With the introduction of periodically driving, we find the picture is similar to the time-reversal symmetric case that when the driving frequency *ω* is much larger than other energy scales, there is a large energy gap between Floquet bands and no edge states. With decreasing the driving frequency, the gap at 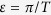 firstly closes and then reopens, with edge states emerging and traversing the reopened gap as shown in Fig. 5(a). This result is similar to the one obtained by periodically varying the parameter *M*^10^. As the system is without TRS, here the edge states are chiral in the sense that the fermionic atoms with opposite velocity propagate on the opposite boundary.

With a further decrease of the driving frequency, the gap at *ε* = 0 will also close and then reopen. As a consequence, chiral edge states traverse both gaps at *ε* = 0 and 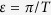. As the winding number of a band is equal to the difference between the number of edge states at the gaps above and below the band, 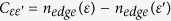, it is direct to see that the two bands’ winding numbers in Fig. 5(b) are both *zero*. This is another unique property of a periodically driving system that the chiral edge states can exist despite the fact that the Chern numbers associated with both bands are zero^40^. These chiral states are localized at the two open boundaries of the system, as shown in Fig. 5(c,d).

**Figure 6:**
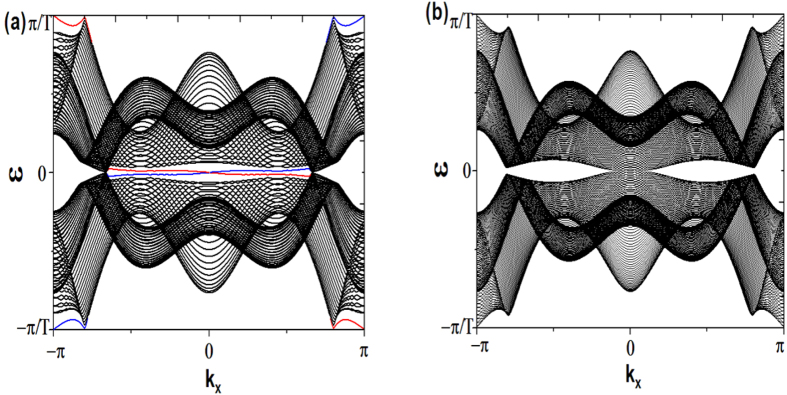
Anisotropic driving cases. (**a**) Driving along the *y* direction, Floquet spectrum with parameters: *J* = 1, *B* = 0.2, *M* = 0.5, 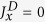, 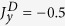, and 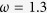. (**b**) Driving along the *x* direction, Floquet spectrum with parameters: *J* = 1, *B* = 0.3, *M* = 0.8, 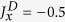, 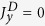 and 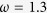.

If we only drive the system along the *y* direction, i.e. 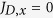, 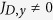, we find the results are similar to the isotropic case’s. Figure 6(a) shows that chiral edge states traverse both gaps, however, compared to the isotropic case under the same parameter condition except 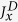, we find the gaps at *ε* = 0 and 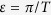 are greatly decreased. If we instead only drive the system in the *x* direction, in other word, 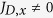, 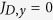, the picture is dramatically changed. No matter what parameters are chosen, there is no edge state emerging, therefore, the system is always a trivial insulator, as shown in Fig. 6(d). Although driving the system along the direction with periodical boundary condition will not induce chiral edge states at the open boundary, such a driving has the effect that it enlarges the energy gap.

The effect of an external harmonic trap to this model is similar to the time-reversal symmetric Kane-Mele model, and therefore, we neglect the discussion here.

## Discussion

As the usual driving approach for solid state systems, which takes the advantage of electromagnetic field, is found to be incompetent to drive cold atomic insulating systems with TRS to be topological, we point out that simply varying the optical lattice potential periodically provides a general and effective way to overcome this. Based on the time-reversal symmetric Kane-Mele model, it is found that this simple approach can effectively drive the system from the trivial phase to the topological phase named as a FQSH insulator. This novel phase can stably host one or two pair of helical edge states at the same boundary. For a static QSH insulator, the simultaneous appearance of two pair of helical edge states is unstable and the edge states are easy to be gapped out. This suggests that the FQSH insulator is not a simple analog of the QSH insulator.

To realize a FQSH insulators, the most challenge part is no doubt to realize the spin-orbit coupling needed^48^,^49^,^50^,^51^,^52^,^53^,^54^,^55^,^56^,^57^,^58^,^59^,^60^,^61^,^62^,^63^,^64^. Once it is realized, as we have checked, all other parameters are well within current experimental realization. For a system without TRS, we have shown that periodically varying the optical lattice potential can also effectively drive the topologically trivial insulator into the topological regimes where chiral edge states emerge, which is similar to the driving approach by using external electromagnetic field. This suggests that the driving approach we propose is a very general one for cold atomic systems.

## Methods

### The first order approximation

The calculation of hopping amplitudes is similar for different lattice structures and its essence can be captured by the one-dimensional case. For a one-dimensional optical lattice whose potential is given as 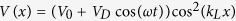, the main property of the on-site wave function can be captured by the wave function 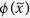 which satisfies the Schrödinger equation: 
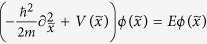
, where 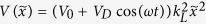 and 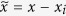 is the relative distance with *x*_*i*_ one of the minima. When the temperature is sufficiently low and the filling number is not bigger than two, the particle will only occupy the lowest energy band and the wave function is given as 
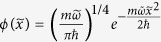
, where 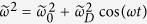 and 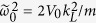, 
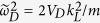
. Based on the wave function, the field operator can be expanded as 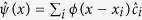, then the nearest-neighbor hopping term is given as


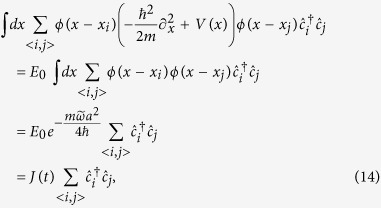


where 
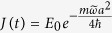
, 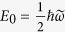, and 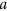 is the lattice length. When 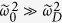, we can expand 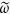 as a series of 
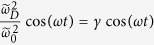
, and then 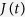 is given as


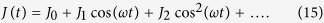


To the second order, the concrete form of the expanding parameters are given as: 
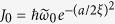
, 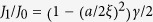, 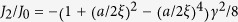, where 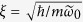. We have checked when the lattice is not very deep, *i.e.*, *a* is only several times larger than 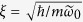, and 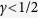, we can safely neglect all terms of 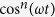 with order 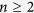 and only keep the 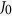 term and 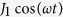 term (in the main text, *J*_0_ and *J*_1_ are denoted by *J* and *J*_*D*_, respectively), in other words, under these two constraints, the first order approximation of 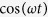 is generally a very good approximation (a clearer picture-illustration is given in the Supplementary information). For the spin-orbit coupling term, the concrete form of the expanding parameters depend on the origin of the spin-orbit coupling. For the Kane-Mele model we consider, if we take the spin-orbit coupling as a pure spin-dependent second-nearest-neighbor hopping, then the first order coefficient 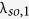 and the second order coefficient 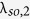 can be obtained from *J*_1_ and *J*_2_ by a substitution of *a* with the distance 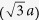 between two second-nearest-neighbor sites, respectively.

We have checked that when 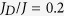, the Kane-Mele model with other parameters taking values the same as the ones in Fig. 1, can already be driven to realize the FQSH insulator with considerable gaps (in the main text, we still take 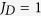 for a better illustration), which suggests that this driving approach is indeed an effective approach.

### Time-reversal symmetry conservation

The Hamiltonian describing a periodically driven system can always be expanded as 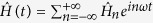, where *n* is an integer. When 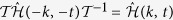 for any given *t*, 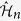 satisfies 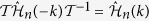 for arbitrary *n*.

The TRS of a Floquet Hamiltonian is defined as: 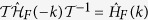, where 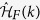 is the Floquet Hamiltonian which is time-independent. 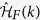 usually has two definitions. The first one corresponds to an effective Hamiltonian with the same matrix size as the static Hamiltonian 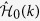, the second one corresponds to an infinite matrix whose concrete form is obtained through Eq.(6). The two definitions are in fact equivalent. If the infinite hermite matrix is diagonalized with diagonal entities to be a matrix with the same size as 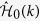, then 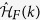 with the first definition can be directly obtained by choosing the matrix entities corresponding to the first quasienergy Brillouin zone, *i*.*e.*, *m* = 0 in Eq.(6). Therefore, if 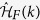 with the second definition holds TRS, 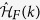 with the first definition also holds TRS. As the concrete form of 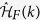 with the first definition is difficult and tedious to obtain, in the following, we focus on discussing the TRS of the Floquet Hamiltonian with the second definition.

As the Floquet Hamiltonian 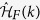 now is an infinite matrix, the matrix form of the time-reversal operator also needs to be generalized to be infinite, *i.e.*, 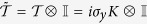 with 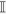 the infinite unit matrix. When 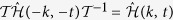, then


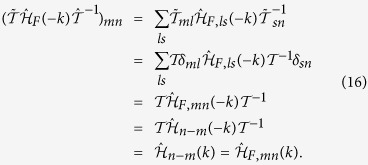


Therefore, 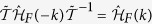, the Floquet Hamiltonian holds TRS. As the expansion of *J*(*t*) in Eq.(15) is composed by a series of 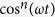 with *n* = 0, 1, 2…, therefore even considering higher order of the expansion, 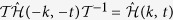 is always hold, which suggests that for the driving approach we propeose, the Floquet Hamiltonian always holds TRS.

For the time-periodic uniform magnetic field given by 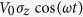 (see Eq.(8)), although 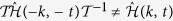, the Floquet Hamiltonian still holds TRS. This can be easy to prove by redefining the time-reversal operator as 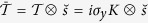, where 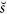 is an infinite matrix whose diagonal entities are given as 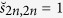, 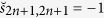. Then following the steps in Eq.(16),


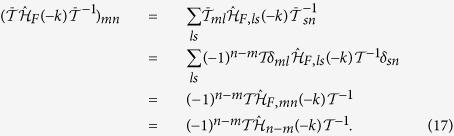


As only 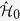 and 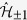 of the Floquet Hamiltonian are non-zero, and 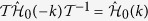 due to the TRS of the static system, 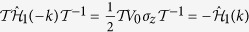, it is direct to find 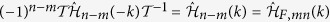, therefore, 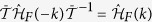, the Floquet Hamiltonian holds TRS.

By using the two kinds of definition of the time-reversal operator, it is direct to verify that all kinds of driving terms of the form 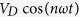 or 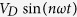 with *V*_*D*_ exhibiting a unique parity under time-reversal operation do not break the TRS of the system. Similar conclusions can also be obtained for *particle-hole symmetry* and *chiral symmetry*.

## Additional Information

**How to cite this article**: Yan, Z. *et al.* A General Time-Periodic Driving Approach to Realize Topological Phases in Cold Atomic Systems. *Sci. Rep.* **5**, 16197; doi: 10.1038/srep16197 (2015).

## Supplementary Material

Supplementary Information
