# Supplementary material for: A General Time-Periodic Driving Approach to Realize Topological Phases in Cold Atomic Systems
Source: Sci Rep. 2015 Nov 6;5:16197. doi: 10.1038/srep16197 (PMC4635429; doi:10.1038/srep16197)
Supplement: Supplementary Information [file srep16197-s1.pdf]

Supplementary Information for a General Time-Periodic Driving Approach to Realize Topological Phases in Cold Atomic Systems

Zhongbo Yan[1], Bo Li[1], Xiaosen Yang[2,3], and Shaolong Wan[1*]
[1]Institute for Theoretical Physics and Department of Modern Physics
University of Science and Technology of China, Hefei, 230026, China
[2]Beijing Computational Science Research Center, Beijing, 100084, China
[3]Department of physics, Jiangsu University, Zhenjiang, 212013, China

*Corresponding author: slwan@ustc.edu.cn

THE VALIDITY REGIME OF THE FIRST-ORDER APPROXIMATION.

Here for simplicity we consider a one-dimensional optical lattice, the two-dimensional case we consider in the manuscript can be directly generalized from it. The lattice potential is given as

$$V(x) = (V_0 + V_D\cos(\omega t))\cos^2(k_L x). \tag{1}$$

To obtain the on-site wavefunction, we expand the potential at its minima as

$$V(\tilde{x}) = (V_0 + V_D\cos(\omega t))k_L^2\tilde{x}^2, \tag{2}$$

here $\tilde{x} = x - x_i$ is the relative distance with x_i one of the minima. Then the Schrödinger equation is given as

$$(-\frac{\hbar^2}{2m}\partial_{\tilde{x}}^2 + V(\tilde{x}))\phi(\tilde{x}) = E\phi(\tilde{x}), \tag{3}$$

which can be rewritten as

$$\frac{d^2\phi}{d\tilde{x}^2} + \frac{2m}{\hbar^2}(E - \frac{1}{2}m\tilde{\omega}^2\tilde{x}^2)\phi = 0 \tag{4}$$

with $\frac{1}{2}m\tilde{\omega}^2 = (V_0 + V_D\cos(\omega t))k_L^2$. By defining $\tilde{\omega}_0^2 = 2V_0k_L^2/m$ and $\tilde{\omega}_D^2 = 2V_Dk_L^2/m$, then $\tilde{\omega}^2 = \tilde{\omega}_0^2 + \tilde{\omega}_D^2\cos(\omega t)$. As there are only two relevant energy scales, $\hbar\tilde{\omega}$, therefore, when $\hbar\tilde{\omega} >> k_BT$, the particles only occupy the lowest energy level. Then

$$\phi(\tilde{x}) = (\frac{m\tilde{\omega}}{\pi\hbar})^{1/4}e^{-\frac{m\tilde{\omega}\tilde{x}^2}{2\hbar}}. \tag{5}$$

Based on this, the field operator can be written as

$$\hat{\psi}(x) = \sum_i \phi(x - x_i)\hat{c}_i, \tag{6}$$

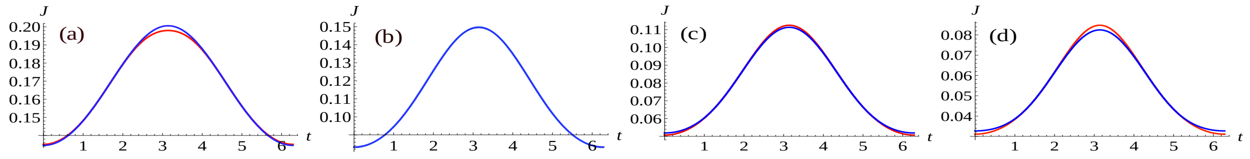

FIG. 1: (color online) $\frac{V_D}{V_0} = \frac{1}{2}$ for all figures. (a) $\kappa = \frac{m\tilde{\omega}a^2}{4\hbar} = 1.8$, (b) $\kappa = 2.2$, (c) $\kappa = 2.6$, (d) $\kappa = 3$. The red line corresponds to $J(t)$, and the blue line corresponds to the expansion of $J(t)/J_0$ to second order of $\cos(\omega t)$. The concrete forms of the expansion are (a) $J_{Exp}/J_0 = 1 - 0.4\cos(\omega t) + 0.01375\cos^2(\omega t) = (1 + 0.006875) - 0.2\cos(\omega t) + 0.006875\cos(2\omega t)$,(b) $J_{Exp}/J_0 = (1 + 0.025625) - 0.3\cos(\omega t) + 0.025625\cos(2\omega t)$, (c) $J_{Exp}/J_0 = (1 + 0.049375) - 0.4\cos(\omega t) + 0.049375\cos(2\omega t)$, (d) $J_{Exp}/J_0 = (1+0.078125) - 0.5\cos(\omega t) + 0.078125\cos(2\omega t)$. It can be seen that under these parameter conditions, the coefficients of $\cos(2\omega t)$ is nearby an order smaller than the coefficients of $\cos(\omega t)$. Furthermore, noting the fact that the coefficients of $\cos(2\omega t)$ enter the matrix form of the Floquet Hamiltonian as $H_{m,m\pm 2}$, which will less effectively affect the eigenvalues of the diagonal part $H_{m,m}$ than $H_{m,m\pm 1}$, which corresponds to $\cos(\omega t)$. Therefore, the second order under these parameter conditions can be safely neglected, and the first order approximation is a very good approximation.

then the model relevant hopping term is given as

$$
\begin{aligned}
&\int dx \sum_{<i,j>} \phi(x-x_i)(-\frac{\hbar^2}{2m}\partial_x^2 + V(x))\phi(x-x_j)\hat{c}_i^\dagger \hat{c}_j \\
&= E_0 \int dx \sum_{<i,j>} \phi(x-x_i)\phi(x-x_j)\hat{c}_i^\dagger \hat{c}_j \\
&= E_0 e^{-\frac{m\tilde{\omega}a^2}{4\hbar}} \sum_{<i,j>} \hat{c}_i^\dagger \hat{c}_j \\
&= J(t) \sum_{<i,j>} \hat{c}_i^\dagger \hat{c}_j,
\end{aligned} \tag{7}
$$

where $J(t) = E_0 e^{-\frac{m\tilde{\omega}a^2}{4\hbar}}$, $E_0 = \frac{1}{2}\hbar\tilde{\omega}$, and a is the lattice length. When $\tilde{\omega}_0^2 >> \tilde{\omega}_D^2$, we can expand $\tilde{\omega}$ as a series of $\frac{\tilde{\omega}_D^2}{\tilde{\omega}_0^2}\cos(\omega t)$, and then $J(t)$ is given as

$$
J(t) = J_0 + J_1\cos(\omega t) + J_2\cos^2(\omega t) + ... \tag{8}
$$

We find that when the lattice is not very deep, *i.e.*, a is only several times larger than $\xi = \sqrt{\hbar/m\tilde{\omega}_0}$, the first order approximation is generally a very good approximation when $\frac{\tilde{\omega}_D^2}{\tilde{\omega}_0^2} = \frac{V_D}{V_0} < \frac{1}{2}$, see Fig.1.

From Fig.1, we can see that J_1/J_0, which is approximately equal to J_D/J, can take values that is not very small compared to 1. We have checked that when $\frac{J_D}{J} = 0.2$, the Kane-Mele model with other parameters taking values the same as the ones in Fig.1 of the main text, can already be driven to realize the FQSH insulator with considerable gaps (in the main text, we still take $J_D = 1$ for a better illustration). Therefore, this driving approach is indeed an effective approach.

Based on the above analysis, now we give a discussion about the experimental realizable values of the parameters in the Kane-Mele model. Without loss of generality, we consider a two-component ^{40}K fermi gas loaded in the hexagonal lattice realized in the experiment [1] whose near-neighbor distance $d = \lambda/3$ with the wavelength of the laser $\lambda = 830nm$. Then the recoil energy $E_R = k_L^2/2m \approx 45$kHz (here $\hbar = 1$). Between two nearest neighbor energy minima, the energy barrier is equal to $V_0/4$, and the expansion of the potential at the neighborhood of the minimum is given as $3V_0k_L^2x^2/16$, where x is the distance to the minimum.Then $\tilde{\omega}_0 = \sqrt{3V_0E_R}/2$. If we take $m\tilde{\omega}_0 d^2/4\hbar = 2$, a direction calculation shows $V_0 \approx 17.8E_R$, $J_0 \approx 0.25E_R$. Then from Fig.1 of the main text, it is direct to find that the frequency ω of the driving terms in the topological regimes is approximately given as E_R. All topology-related parameters except λ_{so} are well within current experimental realization.

* slwan@ustc.edu.cn

[1] Soltan-Panahi, P., Struck, J., Hauke, P., Bick, A., Plenkers, W., Meineke, G., Becker, C., Windpassinger, P., Lewenstein, M. & Sengstock, K. Multi-Component Quantum Gases in Spin-Dependent Hexagonal Lattices, *Nat. Phys.* **7**, 434 (2011).
